# Early-Phase Interventional Trials in Oral Cancer Prevention

**DOI:** 10.3390/cancers13153845

**Published:** 2021-07-30

**Authors:** Caroline McCarthy, Stefano Fedele, Christian Ottensmeier, Richard J. Shaw

**Affiliations:** 1 Liverpool Head and Neck Centre, Institute of Systems, Molecular and Integrative Biology, University of Liverpool, Liverpool L3 9TA, UK; C.Ottensmeier@liverpool.ac.uk (C.O.); rjshaw@liverpool.ac.uk (R.J.S.); 2 Department of Oral Medicine, Liverpool University Dental Hospital, Liverpool L3 9TA, UK; 3 Eastman Dental Institute, University College London, 21 University Street, London WC1E 6DE, UK; Stefano.fedele@nhs.net; 4 National Institute for Health Research, University College London Hospitals Biomedical Research Centre, Maple House Suite A 1st floor, 149 Tottenham Court Road, London W1T 7DN, UK

**Keywords:** oral epithelial dysplasia, chemoprevention, early-phase trials, oral cancer prevention, phase I, phase II, first-in-human

## Abstract

**Simple Summary:**

Oral cancer is a devastating disease with increasing incidence worldwide. Oral epithelial dysplasia (OED) is a potentially malignant disorder and patients with OED are at increased risk of developing oral cancer. Current strategies for management of OED include surgery or close observation and both fail to address the underlying pathogenesis of the disease. There is an urgent need for evidence-based medical treatments for OED to prevent oral cancer development in this cohort. Chemoprevention trials to date have not delivered therapeutic agents for routine clinical practice. Historically, there has been significant heterogeneity in the design of oral cancer chemoprevention trials, with most failing to selectively recruit patients with biopsy-proven OED, which limits the usefulness of the findings in the OED population. The present paper aims to review the current evidence and the methodology of early-phase trials in oral cancer chemoprevention. Novel strategies in oral cancer chemoprevention will also be discussed.

**Abstract:**

The increasing breadth of molecular targets, promise of immune-targeted therapies and repurposed agents have heightened interest in cancer prevention. While, to date, testing of oral cancer chemoprevention strategies has failed to deliver therapeutic agents for routine clinical practice, there remains an urgent need for further clinical research to overcome this hurdle. Patients at the greatest risk of disease stand to benefit the most from inclusion in clinical trials; therefore, there is a need to carefully define this population using validated clinical and molecular markers. Safety, tolerability and the efficacy of interventions is assessed through carefully selected endpoints. These endpoints may include pharmacodynamic, clinical, histological and on-target molecular modifications as an individual or as a composite endpoint. Early-phase trials provide an area of opportunity to explore novel and repurposed agents in the setting of oral cancer chemoprevention, eventually leading to phase III trials with clinical endpoints such as transformation and clinical outcome; these studies are large, lengthy and expensive and should be reserved for the most promising of agents. This paper will explore current evidence in oral cancer chemoprevention, drug repurposing, selection of appropriate endpoints for early-phase trials and novel therapeutic angles in oral cancer chemoprevention.

## 1. Introduction

Approximately 475,000 individuals are diagnosed with oral and oro-pharyngeal cancer worldwide each year [1], with the main risk factors including smoking and smokeless tobacco, betel nut, alcohol, human papillomavirus (HPV) infection and the presence of oral potentially malignant disorders such as oral leukoplakia, lichen planus and oral submucous fibrosis [2,3]. Oral and oro-pharyngeal cancer are characterized by a poor prognosis, with current data suggesting an overall mortality of approximately 47% (225,000 deaths per year) [1].

The current model of oral and oro-pharyngeal cancer development suggests a progressive multi-step transition from normal mucosa to oral squamous cell carcinoma (OSCC) through a series of progressive histological changes (oral epithelial dysplasia) reflecting the accumulation of genetic and epigenetic abnormalities and genetic susceptibility [4,5,6,7]. In patients with oral leukoplakia and other potentially malignant disorders, oral epithelial dysplasia (OED) represents the main predictor of future cancer development [8,9]. Assessment and grading of OED is widely used in clinical practice to inform prognosis and guide treatment: current data suggest that approximately 12% of individuals with OED eventually progress to OSCC, with differential progression rates for mild/moderate and severe dysplasia being 10% and 24%, respectively [8,10]. Management of OED is controversial and there remains no widely accepted standard of care. The main aim of OED treatment is to reduce the risk of/prevent cancer development; however, there is a paucity of robust evidence to support any one strategy. Current management is limited to surgical excision or close surveillance [11], with both strategies failing to address the molecular changes driving disease progression and the development of multiple and multifocal dysplastic and malignant oral mucosal changes (field cancerization), which are well-described characteristics of oral carcinogenesis [4,12,13]. A recent RCT of surgery vs. observation in non-dysplastic leukoplakias failed to demonstrate an effect on cancer outcomes between groups at median follow up of 72 months. In addition, there were higher rates of worsening lesions/recurrence in the surgery group [14]. These findings cannot be extrapolated to dysplastic oral lesions and further work is ongoing by the same group to address this question [14].

Cancer chemoprevention represents an attractive alternative management strategy for OED due to the potential to overcome the above limitations: it can (i) inhibit the process of carcinogenesis and prevent the development of malignancy, (ii) avoid the morbidity associated with surgery and (iii) tackle field cancerization. Furthermore, it may be useful in preventing second primary tumours or recurrences in patients with a history of oral cancer.

Several oral cancer chemopreventative strategies have been explored through pre-clinical studies and clinical trials but none have gained widespread acceptance [15], mostly due to toxicity or lack of efficacy of the proposed agents and inconsistencies with respect to study size, target population, inclusion criteria, outcomes measures and endpoints. Designing a chemoprevention study is indeed challenging as, extrapolating from trials in other precancer/cancer settings, trials should ideally incorporate clinical, histological and molecular endpoints and observe participants for long enough to capture cancer development, and possibly survival, as main clinical outcomes [16].

The present paper aims to review the current evidence and discuss the methodological challenges behind the design of early-phase trials in oral cancer chemoprevention.

## 2. Management of Oral Epithelial Dysplasia: Current Standard of Care

Management of OED remains controversial and not evidence based. Although it is widely accepted that the progression of OED to OSCC occurs only in a small subgroup of patients, there is no robust method to identify these individuals. As a consequence, it remains challenging for clinicians to select the individuals at high risk of progression who would require surgical, potentially morbid, treatment vs. those who may be offered clinical surveillance as they have indolent disease that is unlikely to progress. The current standard of care consists of risk stratification based on histopathological OED grading and a number of clinical factors that have been associated with the risk of OSCC development. Severe OED is associated with a greater likelihood of progression to OSCC (24.1%) compared to those with mild–moderate OED (10.3%) [8]. As a consequence, patients in this high-risk group are often offered surgical excision of the area of OED in the attempt to reduce the risk of future OSCC development. However, the outcomes of surgery in patients with OED vary widely among studies, and there remains little convincing evidence that surgical excision of areas of OED is robustly associated with a reduction in the risk of oral cancer development [17,18]. Furthermore, recurrence of OED [19,20,21] and OSCC development following surgery has been reported in up to 17% and 12% of cases, respectively [17,18,22,23,24,25,26]. Surgical excision may also be justified on the basis that, following an incisional biopsy showing OED, occult OSCC foci can be detected in up to 10% of cases undergoing subsequent surgical excision [27]. Nonetheless, surgery can be associated with significant costs and morbidity, and may not be feasible in all cases due to various clinical and patient factors. In addition, surgery does not address the issue of field cancerization and fails to address the molecular changes driving disease progression.

Patients with mild and moderate OED are usually considered at lower risk of progression [28] and are often offered clinical surveillance (e.g., regular visual inspection) at a variable frequency (from one to four times a year). The rationale is that the likelihood of oral cancer development in these patients is substantially small and does not justify the potential morbidity of surgical interventions. Exceptions include those patients with mild/moderate OED who also present clinical factors associated with progression to OSCC, and are therefore upgraded to high risk and managed accordingly (e.g., non-smoking status, site, non-homogeneous appearance and size of the clinical lesion harbouring OED) [11,28]. Further biopsy is usually provided during surveillance if there is a clinical change in the lesion suggesting possible progression to a higher grade of dysplasia [11]. There are instances, however, where the additional biopsy reveals the presence of an already developed OSCC [29], which suggests that clinical surveillance may notably help with the early detection of high-risk OED or early OSCC, but cannot reduce the risk of OED progressing to malignancy per se.

## 3. Current Evidence in Oral Cancer Chemoprevention

Chemoprevention studies should ideally recruit participants at increased risk of progression to cancer to maximize (i) the event rate (oral cancer development) and (ii) the potential benefit to the patient. Therefore, chemoprevention trials should selectively recruit patients with a prognostic marker associated with a higher risk of oral cancer development, such as OED and/or the presence of LOH at key loci [30,31]. Unfortunately, the vast majority of previous chemoprevention trials have instead recruited individuals at variable risk of progression; for example, patients with a diagnosis of oral leukoplakia with/without OED. The chemopreventative agents used in previous studies (reviewed by Lodi et al.) [15] include vitamin A or retinoids, β-carotene, NSAIDS, herbal extracts, topical bleomycin and a Bowman–Birk inhibitor, for a total of 14 studies and 909 participants (Table 1) [19,21,32,33,34,35,36,37,38,39,40,41,42,43]. These studies included patients with a diagnosis of oral leukoplakia but did not mandate the presence of OED as an inclusion criterion. Only half of the studies (7/14) reported information about the presence of OED at baseline, which ranged from 18 to 73%. Of note, the randomized, placebo-controlled trial by Mallery et al., which investigated the potential chemopreventative effects of freeze-dried black raspberry gel upon the risk of progression to cancer, recruited 40 patients all with a diagnosis of oral intra-epithelial neoplasia (OED or atypia) [34]. Furthermore, the EPOC study recruited patients with leukoplakia considered at high risk of progression due to the presence of loss of heterozygosity (LOH) [44]. Mallery et al. reported a significant reduction in the composite, surrogate endpoint of clinical, histological and LOH profile, after 3 months of therapy between the active and placebo group [34]. The EPOC study found no significant difference in cancer-free survival between intervention (erlotinib 150 mg OD 12 months) and control groups with median follow up of 35 months [44] (see  Section 7.1). The remaining studies reported by Lodi et al. reveal no clinical and/or histological response to chemopreventive interventions in five studies [21,32,35,36,43], high recurrence rates following an initial response in two [33,45] and some evidence of initial clinical response in six of the studies [37,38,40,41,42]. Tsao et al. report some difference in clinical response (reduction in lesion size) after 12 weeks of green tea therapy vs. placebo in hyperplastic and dysplastic oral lesions but no difference in cancer-free survival between groups at 27.5 months [37]. The remaining five studies [38,39,40,41,42] demonstrating a clinical and/or histological response, detailed in Table 1, do not report follow-up data beyond 3 months of cessation of the study treatment, thereby failing to show any evidence of any prolonged treatment effect.

### Drug Repurposing

The complexities, cost, failure rate and time associated with the development of new anti-cancer agents have made drug repurposing an attractive option. Large epidemiological studies have revealed the potential anti-cancer effects of existing drugs by chance, such as aspirin [46,47], metformin [48] and sodium valproate [49]. There is often helpful data detailing the safety profile and pharmacodynamics of established medications, meaning that trials investigating the drug in a new setting can begin at phase II, rather than requiring pre-clinical and phase I studies [50]. The so-called ‘in silico’ method, which uses high-throughput screening to identify candidates for drug repurposing with desired action on a pre-specified target, could help to pre-select drugs which may have a higher chance of ultimate success in phase III trials [51].

## 4. Methodological Challenges in Clinical Trials of Oral Cancer Chemoprevention

### 4.1. Recruitment: Defining the High-Risk Population

The risk of oral cancer development in OED has been associated with a number of clinical, histological and molecular variables. It has been suggested that patients with the highest risk of developing the disease are selectively recruited to clinical trials to maximize the event rate (development of oral cancer) and therefore minimize the number of patients required to detect a difference in treatment effects [44]. This assumes that cancer development is used as the primary outcome.

The mutational landscape of OSCC has been well characterized in recent years, with the use of next-generation sequencing and high-throughput molecular profiling [52,53,54]. The pattern of molecular alterations changes from the premalignant state to the cancer state; however, mutations are detected even early in the process of carcinogenesis [55]. Identification of a panel of mutations which accurately predict oral cancer development may help to identify high-risk patients for targeted recruitment to clinical trials. LOH at key sites (3p14, 9p21) has also been shown to be associated with a higher risk of oral cancer development compared to LOH-negative lesions [30,56]. This has been translated into work in non-smokers with OED which has demonstrated that the combination of non-smoking status and LOH-positivity dramatically increases risk of oral cancer development [57].

Various clinical factors are known to be associated with a higher risk of oral cancer development in OED including size of lesion > 200 mm^2^, heterogenous/speckled appearance (erythroleukoplakia) and lateral tongue sub-site [23,28,58,59]. Clinical factors will therefore remain an important aspect of risk stratification in OED until a biomarker or panel of biomarkers has been validated and adopted into routine clinical practice. Grade of dysplasia (WHO grade mild, moderate, severe) [60] can also be used to stratify patients into higher and lower risk cohorts; moderate and severe oral epithelial dysplasia have been shown to have a greater than two-fold increased risk of malignant transformation compared to mild OED [12]. The issues of sampling error and intra- and inter-observer variability continue to limit the predictive ability of histological grade as an isolated variable [61]; however, its usefulness in clinical practice as a well-established diagnostic tool is recognised.

No individual variable is able to reliably predict the future development of oral cancer in an individual with OED. However, combinations of risk factors can be used to generate a binary high- or low-risk profile which can then be used to guide management strategies for that individual [11].

Selective recruitment of high-risk individuals is advantageous; these individuals stand to gain the most from any intervention and, if cancer development is to be used as an endpoint, it maximizes the event rate. Accurately defining the methodology around selective recruitment and justification for this approach should be clearly reported to allow comparability of trials.

### 4.2. Choice of Endpoints

#### 4.2.1. Pharmacodynamic and Pharmacokinetic

Pharmacodynamic (PD) endpoints are often used in phase 0 and phase I trials to demonstrate proof of mechanism, i.e., the agent engages its intended molecular target resulting in a (prior determined) desired alteration of the target function. This should ultimately result in a cascade of events that leads to a clinical response. Pharmacokinetic endpoints are also used to explore the absorption, distribution, metabolism and elimination of a drug, if this has not been previously defined; establishing the relationship between PK and PD endpoints is important in understanding dose–response [62]. This will usually involve measuring plasma levels of drug at specified time intervals and correlating this with patient drug diaries [63], as in Saba et al.’s study of erlotinib in OED [64].

#### 4.2.2. Safety

Any medicinal product that is to gain regulatory approval must demonstrate a strong safety record. Therefore, recording of adverse events is a requirement of all clinical trials of investigational medicinal products. Adverse events (AEs) represent off-target occurrences that may or may not be associated with the drug or product under investigation. Phase I trials are often solely concerned with safety and may therefore not have a control group, as the aim is not to show efficacy of the drug in question [65]. The aim of phase I trials is often to determine maximum tolerated dose (MTD), which is the highest dose that does not cause unacceptable side effects. The MTD has traditionally been determined via rule-based designs, such as the 3 + 3 or A + B designs [66]. More recently, model-based designs, such as the continual reassessment method (CRM), have gained acceptance as more efficient methods in which a statistical model is used to estimate the relationship between drug dose and disease-limiting toxicity (DLT) risk. Use of the CRM has been shown to be more efficient in determining MTD and ensuring more patients are treated close to MTD, compared to the 3 + 3 design [67]; however, expert statistical support is required. Appropriate stopping rules must also be defined, usually based on number and severity of adverse events, to ensure the safety of trial participants [68].

#### 4.2.3. Surrogate Markers of Oral Cancer Development

##### Clinical

A measure of clinical response is universally reported in clinical trials in OED but the method of defining clinical response varies [15]. Often a grading system is used to categorize changes in size of the dysplastic lesion following intervention, with the changes defined as complete, partial or no response [63,69]. To reduce intra- and inter-variability in measurement of clinical lesions, Mallery et al. stipulated the use of an in-field ruler (Puritan Stick^©^) during clinical photography. This, together with the use of image software, allowed blinded assessment of clinical photographs to determine changes in size of lesion [34], which reduces bias in this measurement. 

##### Histological

The use of ‘complete’ and ‘partial’ response categories has also been applied to histological grading, using change in WHO grade of dysplasia pre- and post-treatment [64].

##### Molecular

Molecular endpoints should be chosen based on good pre-clinical data that have accurately defined the mechanism of action of the drug under investigation; the endpoint should represent an on-target effect of the drug that is also associated with future risk of cancer development [31,32,34,40,41,42,43,44,64,70].

#### 4.2.4. Composite Endpoints

A composite endpoint consists of two or more distinct endpoints, known as components [71]. Both the Mallery trial [34] and the SAVER trial [31] use the same composite surrogate endpoint, which combines clinical, histological and molecular endpoints associated with cancer progression in OED. The benefit of combining endpoints in this way is increased power to detect differences in the primary endpoint due to an increased number of ‘events’; this, in theory, allows smaller trials at lower cost and is attractive in the case of OED where the event rate (oral cancer development) is low. Components of the composite endpoint should all occur with similar frequency, have a clinically meaningful interpretation and similar strength association with the disease in question. As described, clinical factors (size of lesion), histological grade of dysplasia and LOH positivity have all been strongly associated with oral cancer development, which makes this combination of components as a composite endpoint plausible for trials in OED. These interpretations can all be assessed blinded which adds to the credibility of trial design in reducing potential bias from unblinded assessment in open-label trials.

#### 4.2.5. Oral Cancer Development as an Endpoint

Lesions with histological evidence of OED are known to progress to oral cancer in 12% of cases, with a mean time to transformation of 4 years [8]. The annual malignant transformation rate appears to be stratified by grade of dysplasia with a 3.57% rate for severe OED compared to 1.7% for mild OED [72]. Cancer endpoints are considered the gold standard in chemoprevention trials [15,73]; however, the low event rate that would be expected in short-duration trials would necessitate large numbers to demonstrate efficacy, which limits the usefulness of cancer endpoints in this context. Therefore, cancer endpoints are best suited to large phase III trials in drugs with a proven track record in previous early-phase trials.

### 4.3. Window of Opportunity Design

Window of opportunity trials use the time between diagnosis and treatment as an opportunity to explore novel therapeutic strategies, without compromising standard of care [74]. This is an attractive option, as diagnostic biopsies can often serve as the baseline research biopsy and final surgical excision specimens can be sampled for the purposes of collecting endpoint data. That is, it is possible to minimize and, in some cases, eliminate the need for extra invasive samples for the purposes of research. This method is used in the SAVER trial, where, in patients presenting with a new leukoplakia suspicious for dysplasia, a screening biopsy for the trial also serves as the diagnostic biopsy. Patients scheduled for surgery will have a punch biopsy at the time of surgery (at the 4-month point) and those who are to be kept under observation undergo a second biopsy for the purposes of the trial [31]. This window design is suitable for drugs with well-characterized pharmacokinetic profiles but where bioactivity in target tissue is yet to be established; for example, in drug repurposing trials [74].

## 5. The Need for a Core Outcome Set in OED Trials

The lack of chemoprevention trials in OED limits the ability to synthesize evidence in meta-analysis and therefore limits the quality of the evidence base. The COMET initiative was established in 2010 to promote the development of Core Outcome Sets (COS) in clinical trials [75]. These COS are an agreed set of outcome measures that must be reported in clinical trials within the specified disease. The outcomes included are usually determined via a Delphi process and should include the patient perspective. The COS is a basic panel of outcome measures, all of which must be reported; however, it is recognized that trials should also record endpoints specific to the agent under investigation, such as on-target molecular effects. There is no COS for clinical trials in OED and this should be addressed to ensure the comparability of future studies in this area.

## 6. Initiatives in Oral Cancer Chemoprevention

There is recognition that achieving better outcomes in cancer prevention and treatment involves significant investment and collaboration. The UK Experimental Cancer Medicines Network (ECMC) was established in 2007 through joint investment by CRUK and NIHR and health departments of the devolved nations, with the aim of assisting in the delivery of early-phase cancer studies. Along with significant funding to support cancer prevention research, the ECMC have published consensus guidelines on the effective delivery of complex innovative design trials, which may be of relevance in the oral cancer chemoprevention setting [76]. Oral cancer prevention strategies are high on the agenda for the World Health Organisation (WHO) [77], with a global oral health action plan promised by 2023 and for the International Association for Research on Cancer (IARC), who plan to publish an Oral Cancer Prevention handbook, with a strong focus on tobacco cessation [78]. Similarly, the National Cancer Institute supports research into oral cancer with around USD 20 million of funding per annum and their Division of Cancer Prevention has a subdivision focused on supporting phase 0/I/II trials, the ‘Consortia for Early Phase Prevention Trials’; the NCI is currently supporting an early-phase trial of chemoprevention of oral cancer using a mucoadhesive fenretinide patch in healthy volunteers [79].

## 7. Novel Strategies in Oral Cancer Chemoprevention

### 7.1. EGFR Inhibitors

The EPOC trial was a randomised, placebo-controlled, double-blind multi-centre trial of erlotinib (an EGFR inhibitor) in the oral premalignancy setting, reported in 2016 [44]. Patients were eligible for randomisation if they had an oral premalignant lesion(s) with or without history of oral cancer and evidence of LOH at key loci known to be associated with progression to oral cancer [30]; the ‘LOH positive group’. Patients who did not have evidence of LOH on screening (‘LOH negative group’) were not eligible for randomization and did not receive any intervention but were followed up to assess incidence of malignant transformation. Randomised patients (LOH-positive group) received either erlotinib 150 mg once daily or matched placebo for 12 months. A total of 75 patients were recruited to each group with a median follow up time of 35 months. There was no difference in cancer-free survival in the treatment and control groups after 12 months of treatment with erlotinib (HR 1.27; 95% CI, 0.68–2.38). However, when the LOH-positive group was compared to the LOH-negative group, the former had significantly lower 3-year cancer-free survival (HR 2.19; 1.25–3.83), which demonstrates the potential utility of LOH as a predictive biomarker in OED. This trial was the first chemoprevention trial in OED to selectively recruit patients based on risk of cancer using a biomarker (LOH), signifying a move towards a more personalized approach to clinical trial recruitment.

### 7.2. MET-Targeted Therapy

Santigny et al. reported a microarray gene expression study of 86 leukoplakia samples which identified 26 transcripts associated with increased risk of oral cancer development [80]. Subsequently, the MET oncogene, a tyrosine kinase hepatocyte growth factor (HGF) receptor, was explored in a preclinical (murine model) study, which demonstrated an association between high MET expression levels and oral cancer development [81]. Crizotinib (a multikinase inhibitor with activity against MET [82]) was shown to reduce the frequency of progression from dysplasia to cancer in the mouse model. Therefore, MET activation may be a potential early driver in oral premalignancy and therefore a possible target for future chemoprevention studies. 

### 7.3. Epigenetic Therapies

The term epigenetics is used to explain the connection between the genotype and the phenotype of an organism. It is usually referred to as a genomic mechanism that reversibly influences gene expression without altering DNA sequences. Examples of epigenetic modifications include DNA methylation, histone modifications, chromatin remodelling and the effects of non-coding RNA [83]. Epigenetic changes are highly variable within different cells and tissues and changes are reversible. DNA hypermethylation at key promotor sites (CDKN2A and CDKN2B) is strongly associated with HNSCC and has been observed in precancerous oral tissues, meaning this could be an early event in oral carcinogenesis [84]. Histone acetylation is associated with a relaxed and open chromatin structure which allows transcriptional activity [83]. Therefore, histone deacetylation is associated with a transcriptionally repressive environment which can lead to tumorigenesis. Further, histone modifications appear to occur at an early stage and accumulate during tumorigenesis [85]. Various histone deacetylases are overexpressed in HNSCC and this has led to interest in HDAC inhibitors as therapeutic agents [86]. There are HDACs approved for use in haematological malignancies and limited evidence in combination therapy for some solid tumours [83]. The Kang study demonstrated a possible preventive effect of sodium valproate (a short-chain fatty acid inhibitor of class I and II HDACs) in HNC [49]. It is a well-established drug with a proven safety record in epilepsy and psychiatric disease. Both time- and dose-dependent reductions in the incidence of HNC were demonstrated (HR 0.57 95%CI 0.39–0.85); in addition, there is a plausible mechanism of action for cancer prevention via HDAC inhibition, and therefore this is an attractive option for chemoprevention trials in oral cancer. The SAVER trial is a multi-centre, randomised controlled trial of SV in oral epithelial dysplasia in the UK and aims to recruit 110 participants to SV 500 mg BD or observation arm [31]. This is a window of opportunity trial; patients randomised to the treatment arm will take SV for 4 months, at which point the surrogate end points will be captured and patients will revert to their pre-determined management pathway (surgery or close surveillance). The feasibility of conducting such research will be explored through monitoring recruitment rates and via an embedded qualitative study to explore patients’ perceptions of the trial; both those who accepted and declined to participate. A mechanistic sub-study will also explore the mechanism of action of SV in the OED setting.

### 7.4. Photoactivated Therapy

Photodynamic therapy (PDT) using systemic photosensitisers, such as FOSCAN, has been explored with some good evidence of efficacy in early-stage oral cancers, and is reviewed elsewhere [87]. However, patients were light-restricted for up to 6 weeks post treatment and required a 7-day inpatient stay [88,89]. Some experienced significant pain at the site of treatment and most required opioid analgesics for 1–2 weeks post treatment, with 4–6 weeks healing time [88,89]. Therefore, conventional PDT does not appear to offer significant advantages over surgery for early oral cancers in terms of morbidity or patient experience. Topical PDT using 5-aminolevlunic acid and either laser or LED light source has demonstrated good results in small studies [90,91] and a recent systematic review of 27 relevant studies concluded that PDT appears to offer advantages for oral potentially malignant lesions refractory to other treatments. In addition, side effects of topical PDT are mild and it remains an option for patients who refuse traditional surgical interventions [92]. LightOx, a commercial partnership invested in developing light-based therapies for oral cancer, have developed a novel class of small-molecule, topically applied therapies, with phototoxic properties combined with hydroxamic acid to increase solubility [93]. The compound is topically applied to the oral lesion and activation is performed using a dental curing light, making this potentially suitable for application to OED lesions in an outpatient setting. The topical nature makes systemic absorption unlikely; therefore, there will be no lasting light sensitivity, as with conventional PDT. If the treatment is well tolerated and effective, this would be a useful treatment modality for patients with lesions not amenable to resection and could be used through repeat applications for patients with recurrent or multiple lesions.

### 7.5. Immune Modulation in Oral Epithelial Dysplasia

#### 7.5.1. Tumour Vaccines

Harnessing the immune system for cancer prevention is not a new concept; however, there is growing interest in the development of non-viral cancer vaccines targeted against tumour-specific and tumour-associated antigens [94,95]. The aim is to generate long-term memory T-cell responses and make cancer cells more visible to the immune system, which will then be activated at the earliest point in the process of carcinogenesis if or when that occurs. Phase I and II trials are underway in the HNC setting and are described by von Witzleben et al. [95]. Vaccinations appear to be most effective in this context prior to disease initiation, in healthy individuals. This demonstrates the need to accurately identify those at greatest risk of disease (oral cancer), prior to the development of OED. The population of patients with OED would provide an ideal cohort for trials of cancer vaccines in oral cancer prevention.

#### 7.5.2. Immune Checkpoint Inhibitors

Immune checkpoint inhibitors, e.g., nivolumab, appear to represent a significant breakthrough in the treatment of cancer, with evidence of improved overall survival in oral cancer [96]. The aim of blocking PD-1-dependent immune checkpoints is to improve the T-cell response against tumour cells and therefore reduce cancer-driven local immune suppression. Whilst clinical evidence in OED is not yet reported, there is pre-clinical evidence in a murine model which demonstrates that anti-PD-1 antibody reduced the formation of OED lesions and prevented progression to OSCC [97]. A summary of current immunotherapy clinical trials in OED is shown in Table 2, along with other ongoing or recently completed oral cancer chemoprevention trials.

## 8. Conclusions

There is an opportunity to explore the use of novel and repurposed drugs for oral cancer prevention through careful design of early-phase clinical trials. There is a need for development of core outcome sets in OED trials to reduce heterogeneity and ensure comparability for data synthesis in future meta-analyses. Molecular-based approaches are required to identify high-risk patients for recruitment to early-phase trials. These trials should focus on drugs with a proven mechanism of action in phase 0 and phase I studies and should aim to recruit patients with biomarkers of future disease that are associated with the mechanism of action of the drug.

The oral cavity offers a unique opportunity for progress in early-phase chemoprevention trials of both systemic and topical treatments; it is accessible for longitudinal clinical monitoring of lesions and for cellular or tissue sample collection. Patients with OED are at high risk of developing oral cancer compared to the general population; therefore, recruitment of this cohort to well-designed, prospectively registered clinical trials with strategies that aim to prevent the development of oral cancer should form an important part of the overall oral cancer prevention strategy.

## Figures and Tables

**Table 1:** Summary of randomised controlled trials from Lodi et al. (Information on number of patients with confirmed OED is reported where this information was available in the published study.) PC—placebo controlled; DB—double blind; SB—single blind; BD—twice daily; TDS—three times daily; QDS—four times daily; OPML—oral premalignant lesion; FU—follow up.

Author (Ref)	Year	Intervention	Participants (n)	Trial Design	Inclusion	Primary Outcome Measure	Results
Armstrong [32]	2013	Bowman–Birk inhibitor concentrate (BBIC) 3 g BD	132 recruited 89 completed	Phase IIb, 2-arm, PC, DB, block randomisation, RCT.	Histologically confirmed oral leukoplakia and/or erythroleukoplakia.	Change in lesion area at 6 months	No significant difference in response rate (*p* > 0.94)
Hong [33]	1986	13-cis-retinoic acid (1 to 2 mg/kg/d) for 3 months + 6 months FU	44 randomised 40 completed	2-arm, PC, RCT	Histologically confirmed oral leukoplakia (27% dysplasia)	Clinical and histological response	Clinical: CR or PR in 16/24 retinoic acid vs. 2/20 placebo (*p* < 0.001); histological response in 13/24 vs. 2/10 placebo (*p* = 0.01). Recurrence within 2-3 months of cessation
Mallery [34]	2014	Freeze-dried black raspberries (topically applied gel) 0.5g QDS for 3 months	N = 40	2-arm, PC, DB, RCT	Microscopically confirmed premalignant oral epithelial lesions (72.5% were dysplasias)	Composite surrogate (clinical, histological and LOH markers)	9/22 BRB group high or intermediate response vs. 0/18 placebo (*p* = 0.004). Recurrence in 6/22 BRB and 7/17 placebo at 3 months
Mulshine [35]	2004	Ketorolac 0.1% mouthwash 10mL 30 seconds BD for 90 days vs. placebo	N = 57	2-arm, RCT, PC; DB, randomised 2:1.	Measurable oral leukoplakia	Clinical response	Complete or partial response: 11/37 ketorolac vs. 6/19 placebo (*p* = 0.89)
Papadimitrakopolou [36]	2008	Oral celecoxib 100 mg (arm a) or 200 mg (arm b) or placebo (arm c) BD for 12 weeks	N = 46	3-arm, RCT, PC; DB; randomised 1:1:1.	Histologically confirmed early or advanced oral pre-malignant lesion	Clinical response	No statistically significant differences between the arms were observed in any of the response categories (*p* > 0.05)
Tsao [37]	2009	Green tea 500, 750 or 1000 mg/m^2^ or placebo TDS 12 weeks	N = 39	Phase II, PC, DB dose-finding.	≥1 OPML with at least one high-risk feature (size/dysplsaia/site/pain)	Clinical and histological response	Clinical response: treatment arms 50% vs. 18.2% placebo (*p* = 0.09); histological response 21.4% treatment arms vs. 9.1% placebo (*p* = 0.65). No difference in cancer-free survival between groups at median 27.5 months follow up
Sankaranaryan [45]	1997	Retinoids (vit A 300,000 IU/week) vs. β-carotene (360 mg/week) vs. placebo	N = 131	3-arm, DB, PC RCT	Oral leukoplakia	Cancer incidence Clinical response	Complete resolution: 22/42 vit A, 15/46 β-carotene, 3/46 placebo; *p* < 0.05. Recurrence in 64% vit A and 53% β-carotene after 1 year of cessation
Singh [38]	2004	β-carotene: Group A 4mg BD; Group B 2md BD; Group C placebo; all 3 months	N = 58	3-arm; PC; SB (pathologists)	Not reported (59% had confirmed OED)	Clinical and histological response	Clinical: complete response in 11/20 Arm A; 5/20 Arm B, 0/20 Placebo. (*p* < 0.001); Group A and B showed positive histological response cf placebo (*p* < 0.05). No follow up data
Stich [39]	1988	Retinoids: Group A Placebo; Group B: 200,000 IU/week vitamin A. 6 months.	N = 54	2-arm, PC; unblinded	Oral leukoplakia	Clinical (remission of lesion and development of new lesions) and histological	Remission in 1/33 placebo and 12/21 vitamin A at 6 months Histological changes only assessed in vitamin A group. No FU data on recurrence
Piattelli [40]	1999	Isotretinoin 0.1% gel TDS vs. placebo; 4 months.	N = 9	Cross-over, DB, PC.	Biopsy-proven oral leukoplakia	Clinical response	9/9 had ≥ 50% improvement in size of lesion with active treatment at 4 months. No further FU data
Li [41]	1999	Mixed Tea capsules (0.38 g tea); 2 capsules QDS AND mixed tea in glycerin (10%) applied topically TDS 6/12	N = 59 (age range 23–28 y)	DB, PC, RCT	Clinical diagnosis of oral leukoplakia (20% were dysplasias)	Clinical response (number and size of lesions)	Partial regression in 11/29 (38%) of active treatment arm vs. 3/30 (10%) in placebo at 6 months; *p* < 0.05. No further FU data
Sun [42]	2010	Chinese herbal medicine (ZengShengPing 1.2g TDS) vs. placebo 8-12 months	N = 112	2-arm, SB, PC, RCT	Clinical diagnosis of oral leukoplakia	Clinical response	Clinical improvement in 40/59 treatment gp vs. 9/53 placebo p < 0.01, assessed at 3 months following cessation of treatment
Epstein [21]	1994	Bleomycin 1% applied topically for 5 mins vs. placebo for 2/52	N = 22	DB, PC, RCT	Histologically confirmed oral leukoplakia (22% were dysplastic)	Clinical and histological Response	Mean reduction in size: bleomycin group 81% vs. placebo 21% (*p* = 0.001); no significant difference in change in OED grade between groups. Mean FU 15 months bleomycin and 22 months placebo
Nagao [43]	2015	Intervention: 10mg β-carotene + 500 mg vitamin C vs. active placebo (500 mg vitamin C for 12 months)	N = 46	DB, PC, RCT	Histologically confirmed oral leukoplakia (28% dysplasias)	Clinical response Oral cancer development	Response rate: 17.4% intervention vs. 4.3% placebo. *p* = 0.346. No difference in cancer endpoints

**Table 2:** Summary of oral cancer chemoprevention trials, recruiting or recently completed.

Title	Intervention	Trial Design	Primary Outcome Measure	Location	Number of Participants; OED Inclusion Criteria	Other Info/Status
This Study is to Evaluate the Safety and Pharmacokinetics of SBS-101 in Patients With Oral Premalignant Lesions (NCT03939364)	Isotretinoin oral adhesive film 0.1% vs. 0.2% vs. 0.3% vs. placebo	Phase I, double-blind, placebo-controlled, dose escalation study	Overall response (complete or partial response: clinical or histological) Treatment of emergent adverse events	USA	24; any grade of histologically confirmed dysplasia	Not yet recruiting
A Randomized Study of Sulindac in Oral Premalignant Lesions (NCT00299195)	Suldinac 150 mg PO BD 24 weeks vs. placebo	Randomised, double-blind, placebo-controlled trial	Clinical and histological response	USA/ India	63; dysplasia, any grade	Completed (awaiting results)
Metformin Hydrochloride in Preventing Oral Cancer in Patients With an Oral Premalignant Lesion (NCT02581137)	Metformin extended-release QDS for 2 weeks then BD for 10-12 weeks	Phase IIa; single-group, open-label clinical trial	Clinical response (lesion size)	USA	26; any grade of dysplasia (includes hyperplasia not associated with trauma)	Not yet recruiting
Rosiglitazone Maleate in Treating Patients with Oral Leukoplakia (NCT00369174)	Rosiglitazone maleate 8 mg OD 12 weeks	Phase II, single arm, open label	Clinical or histological response	USA	25; any grade of dysplasia or hyperplasia at a high-risk site	Completed (awaiting results)
Pioglitazone Hydrochloride in Preventing Head and Neck Cancer in Patients with Oral Leukoplakia (NCT00099021)	Pioglitazone Hydrochloride once daily for 12 weeks	Phase II, single arm, open label	Patient’s overall (clinical and histological) response	USA	21; includes hyperplasia at high-risk oral sites or dysplasia at any oral site	Completed (awaiting results). Terminated early due to good results
Safety and Efficacy of Nivolumab in Treating Oral Proliferative Verrucous Leukoplakia (NCT03692325)	Nivolumab IV infusion day 1 of 28 day cycle; up to 4 cycles	Phase II, single group/open-label clinical trial	Best overall response rate (time frame: 2 years)	USA	33; histologically confirmed PVL with any grade of dysplasia	Recruiting
Pembrolizumab in Treating Participants with Leukoplakia (NCT03603223)	Pembrolizumab IV infusion every 3 weeks for 6 months	Phase II, single-arm, open-label clinical trial	Clinical response at 6 months (% patients with complete or partial response)	USA	26; moderate or severe OED	Recruiting
Immune Checkpoint Inhibitor In High Risk Oral Premalignant Lesions: IMPEDE (NCT04504552)	Avelumab 800 mg IV; 4 cycles over 8 weeks	Phase II, single arm, open label	Recurrence free survival/malignant transformation free survival up to 30 months and change in LOH status at 6 months	Italy	240; high risk oral premalignant lesions (with LOH)	Recruiting
Sintilimab to Prevent High-risk Oral Premalignant Lesion Cancerization: STOP (NCT04065737)	Sintilimab 8 cycles over 6 months	Phase II, open label, single arm	Oral Cancer incidence rate at 2 years/clinical response of OPM lesions	China	29; high-risk OED	Not yet recruiting
Vandetanib in Preventing Head and Neck Cancer in Patients with Precancerous Head and Neck Lesions (NCT01414426)	Vandetanib QDS 6 months	Phase II, randomised, double-blind, placebo-controlled trial	Change in microvessel density (MVD) score following treatment	USA	20; any grade of dyplasia plus one “high risk” feature, e.g, LOH at 3p or 9p	Completed (awaiting results)
Sodium valproate for epigenetic reprogramming in the management of high risk oral epithelial dysplasia (SAVER)	Sodium valproate 500 mg BD 4 months vs. observation only	Phase II; open-label, randomised clinical trial	Composite surrogate endpoint: change in clinical, histological and LOH score at 4 months	UK	110; any grade of dysplasia, if mild must have 1+ high risk feature	Recruiting
